# Endometrial hyperplasia with loss of APC in a novel population of *Lyz2*-expressing mouse endometrial epithelial cells

**DOI:** 10.1093/carcin/bgac101

**Published:** 2022-12-22

**Authors:** Susan M Kitchen-Goosen, Heather Schumacher, Julie Good, Amanda L Patterson, Elissa A Boguslawski, Richard A West, Bart O Williams, Galen Hostetter, Dalen W Agnew, Jose M Teixeira, Arthur S Alberts

**Affiliations:** Department of Cell Biology, Van Andel Institute, 333 Bostwick NE, Grand Rapids, MI 49503, USA; Department of Cell Biology, Van Andel Institute, 333 Bostwick NE, Grand Rapids, MI 49503, USA; Department of Cell Biology, Van Andel Institute, 333 Bostwick NE, Grand Rapids, MI 49503, USA; Department of Obstetrics, Gynecology and Reproductive Biology, College of Human Medicine, Michigan State University, Grand Rapids, MI, USA; Department of Cell Biology, Van Andel Institute, 333 Bostwick NE, Grand Rapids, MI 49503, USA; Department of Cell Biology, Van Andel Institute, 333 Bostwick NE, Grand Rapids, MI 49503, USA; Department of Cell Biology, Van Andel Institute, 333 Bostwick NE, Grand Rapids, MI 49503, USA; Department of Cell Biology, Van Andel Institute, 333 Bostwick NE, Grand Rapids, MI 49503, USA; Department of Pathobiology and Diagnostic Investigation, Veterinary Diagnostic Laboratory, College of Veterinary Medicine, Michigan State University, East Lansing, MI, USA; Department of Obstetrics, Gynecology and Reproductive Biology, College of Human Medicine, Michigan State University, Grand Rapids, MI, USA; Department of Cell Biology, Van Andel Institute, 333 Bostwick NE, Grand Rapids, MI 49503, USA

## Abstract

Loss of heterozygosity and promoter hypermethylation of *APC* is frequently observed in human endometrial cancer, which is the most common gynecological cancer in the USA, but its carcinogenic driver status in the endometrial epithelium has not been confirmed. We have identified a novel population of progenitor endometrial epithelial cells (EECs) in mice that express lysozyme M (LysM) and give rise to approximately 15% of all EECs in adult mice. LysM is a glycoside hydrolase that is encoded by *Lyz2* and functions to protect cells from bacteria as part of the innate immune system. Its expression has been shown in a subset of hematopoietic stem cells and in specialized lung and small intestinal epithelial cells. Conditional deletion of *Apc* in LysM + EECs results in significantly more epithelial cells compared to wild-type mice. At 5 months of age, the *Apc*^*cKO*^ mice have enlarged uterine horns with pathology that is consistent with endometrial hyperplasia with cystic endometrial glands, non-villous luminal papillae and nuclear atypia. Nuclear accumulation of β-catenin and ERα, both of which are known to induce endometrial hyperplasia, was observed in the EECs of the *Apc*^*cKO*^ mice. These results confirm that loss of APC in EECs can result in a phenotype similar to endometrial hyperplasia.

## Graphical Abstract

**Graphical Abstract:**
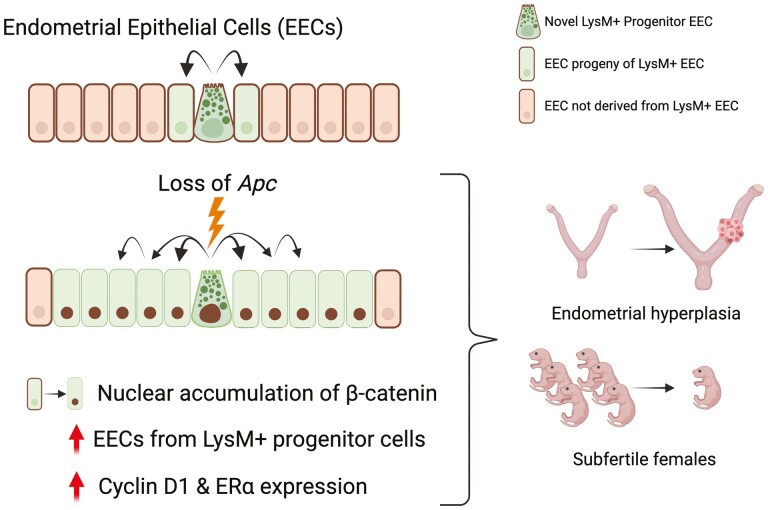

## Introduction

Adenomatous polyposis coli (APC) is a tumor suppressor protein that functions in cell migration, cell adhesion, proliferation and differentiation through both its role in canonical WNT signaling and its interactions with the cytoskeleton (1). APC suppresses the WNT signaling pathway by aiding in the destruction of β-catenin and thereby inhibiting the activation of downstream targets that function in cell proliferation and differentiation (2,3). WNT signaling plays an important role in uterine development and function (4–10), and mouse models in which the WNT signaling pathway is activated specifically in the uterus (via loss of APC or expression of a stabilized form of β-catenin) result in uterine defects including myometrial defects, adenomyosis, endometrial hyperplasia, endometrial cancer and infertility (11–14).

Components of the WNT signaling pathway are frequently mutated in endometrial cancers. Mutations in exon3 of β-catenin that result in accumulation of protein in the cytoplasm and nucleus are seen in 11–44% of endometrial cancers (15–20). Furthermore, these mutations seem to occur in type I endometrioid endometrial cancers, as opposed to type II endometrial cancers (18,21). APC is not commonly mutated in endometrial cancers, although hypermethylation of the APC promoter 1A has been observed in approximately 45% of endometrial cancers (18,19,22). Although these reports indicate that loss of APC and subsequently increased β-catenin can be correlated with endometrial cancer, loss of APC as an actual driver of carcinogenesis has not been shown.

Lysozyme M (LysM) is an enzyme encoded by *Lyz2* gene that functions in the innate immune system by catalyzing the hydrolysis of glycosidic bonds in bacterial cell walls (23,24). LysM has more recently been defined as a marker for myeloid lineage cells, specifically macrophages and granulocytes (25). A subset of hematopoietic stem cells (HSCs) that are capable of long-term repopulation of the hematopoietic system and interventricular septum precursor cells during embryonic development also express LysM (26,27). Additionally, LysM and a second isoform, lysozyme P (LysP), are expressed in specialized epithelial cells in the lungs (type II alveolar epithelial cells) (28) and small intestinal Paneth cells (25). Innate immunity is also a component of the endometrium with leukocyte-derived lysozyme contributing protection against uterine infection (29), but LysM expression has not been characterized in the endometrial epithelium.

Here we describe a novel subset of endometrial epithelial cells (EECs) that are characterized by LysM expression. These cells account for approximately 1% of all endometrial epithelial cells. Using lineage-tracing experiments, we found that the LysM + EECs give rise to 15% of all EECs in mice, and loss of APC in the LysM + EECs resulted in endometrial hyperplasia, a precursor for endometrial cancer. These data suggest that LysM + EECs are progenitor cells for a subset of the endometrial epithelium and are capable of initiating tumor formation. Understanding how the proliferation of LysM + EECs is controlled will help to treat proliferative diseases of the endometrium, such as endometrial cancer and endometriosis.

## Materials and methods

### Mouse models

The Van Andel Research Institute Institutional Animal Care and Use Committee approved all animal experiments. Mice were housed four to five per cage in a room with 12-h light/dark cycle with access to food and water *ad libitum*. LysM-EGFP knock-in mice (30) mice were obtained from Mutant Mouse Regional Resource Centers (MMRRC) (strain name: B6.129(Cg)-*Lyz2*^*tm1.1Graf*^/Mmmh MMRRC ID#: 012039). LysM-Cre knockin (B6/129) mice (31) mice were obtained from Jackson Labs (strain name: B6.129P2-*Lyz2*^*tm1(cre)Ifo*^/J; stock number: 004781) and were crossed with *Apc*^*flox/flox*^ (B6/129) (32). mT/mG reporter mice were obtained from Jackson labs (strain name: B6.129(Cg)-*Gt(ROSA)26Sor*^*tm4(ACTB-tdTomato,-EGFP)Lu*^/J; strain number 007676). Littermates were used as controls for any given genotype. All genotyping was done by PCR of tail biopsy DNA as described in the above references.

### CBC and bone marrow analyses

Peripheral blood was collected by cardiac puncture and complete blood count analysis was performed on a VetScan HM2 Hematology System (Abaxis). Bone marrow was collected by flushing femurs with 3 ml of PBS using a syringe and fine gauge needle. Briefly, red blood cells were lysed, and the remaining cells were washed in PBS with 1% sodium azide and fixed in 1% methanol-free formaldehyde in PBS containing 0.1% FBS. A minimum of 10 000 events were acquired. Flow cytometric analyses were performed with either a FACSCalibur 4-color or a FACSAria 12-color flow cytometer (Becton Dickinson). Data were analyzed using Becton Dickinson CellQuest Pro and FACSDiVa software.

### Estrous staging

Vaginal cytology was done to assess the stage of estrous cycle (33). Twenty microliters of phosphate-buffered saline was injected via pipette into the vagina and flushed 3–5 times with same PBS. The final flush was collected in the pipette tip, placed on a slide, and visualized by light microscopy to determine the stage of estrous of each mouse.

### Tissue preparation and histopathology

Mice were weighed and checked for gross abnormalities at the time of necropsy. Uterine horns were removed, weighed and fixed in 10% (vol/vol) neutral buffered formalin (NBF) along with the whole reproductive tract (ovaries, cervix and vagina). The reproductive tract was embedded in paraffin and 5 µm sections were cut and stained with H&E Discovery XT System from Ventana Medical Systems and analyzed by a pathologist who specializes in the female reproductive tract.

### Immunohistochemistry and immunofluorescence

Immunohistochemistry was performed on formalin-fixed, paraffin-embedded 5 µm sections using the Discovery XT System from Ventana Medical Systems. B-catenin antibody (Cell Signaling #9562 1:200), Cyclin D1 antibody (Cell Signaling #2978) and ERα antibody (Santa Cruz #sc542 1:100). For Immunofluorescence of uteri from *Apc*^*cKO*^ and mT/mG mice, tissues were harvested and fixed in either 10% NBF or 4% PFA then incubated in 15% sucrose overnight at 4°C. Tissues were embedded in either 7.5% gelatin/15% sucrose solution or O.C.T. Compound (Fisher Scientific 23-730-571), embedded on a cold block and frozen at −60°C in isopentane. Seven-micron sections were cut and gelatin was removed by incubating the slides in PBS for 10 min at 37°C. Tissue sections from mT/mG mice were counter stained with DAPI mounting medium and cover slipped. *Apc*^*cKO*^ tissue sections were incubated with lysozyme and cytokeratin antibodies (DAKO #A009902, Developmental Studies Hybridoma Bank (DSHB) TROMA-III CK19 1:50) overnight at 4°C. Tissues were incubated with secondary antibodies (Alexa Fluor 488 goat anti-rat IgG, 1:500 and Alexa Fluor 594 goat anti-rat IgG, 1:500; Life Technologies) for 1 h at room temperature, counterstained with DAPI mounting medium and cover slipped. Urogenital ridges were isolated from seven female LysM-cre; mTmG mice. Seven-micron sections were taken through the whole tissue and Müllerian ducts were analyzed for GFP expression. Uteri from four LysM-cre; mTmG mice at PND7 were analyzed for GFP expression.

### Uterine single-cell suspensions and flow cytometry

Uteri were removed from mice, cut longitudinally, and then cut into ~0.25 cm sections and placed in 10× Gentle Collagenase/Hyaluronidase (Stem Cell Technologies #07919) diluted 1:10 in DMEM F12 (Gibco #12634-010) for 4 h at 37°C with intermittent vortexing. Digested uteri were centrifuged for 5 min at 450*g*, resuspended in ammonium chloride (Stem Cell Technologies #07800) to lyse the red blood cells, and washed with PBS. Digested uteri were centrifuged again, resuspend in Trypsin EDTA (Stem Cell Technologies #07901), triturated prior to adding HBSS + 2% FBS (HF), recentrifuged and resuspended in Dispase with 1 mg/ml DNaseI (Stem Cell Technologies #07913 & #07900) by trituration. Cells were resuspended in HF and 0.1 mg/ml DNaseI and incubated with EpCAM (Biolegend #118217), CD11b (TONBO Biosciences #20-0112) and Thy1 (Biolegend #105325) antibodies for 30 min at 4°C in the dark. After staining cells were washed two times in HF and resuspended in HF with DNaseI with DAPI. Data was acquired using Beckman Coulter MoFlow Astrios and at least 500 000 events were collected in the live, single-cell gates for experimental tubes and 200 000 events for control tubes. Fluorescence minus one controls were used to set gates and single color tubes for both antibodies and endogenous fluorescence were used for compensation. Data were analyzed using Beckman Coulter Kaluza Analysis software.

### Serum collection for estrogen and progesterone analysis

Blood was collected from 15 littermate control and 15 *Apc*^*cKO*^ 8-week-old random cycling female mice via retro-orbital bleed. Serum was isolated using BD Vacutainer Serum Separator tubes. Estrogen and Progesterone levels were assayed in duplicate via ELISA by the University of Virginia Center for Research in Reproduction Ligand Assay and Analysis Core.

## Results

### LysM is expressed in ~1% of EECs

**Figure 1.**
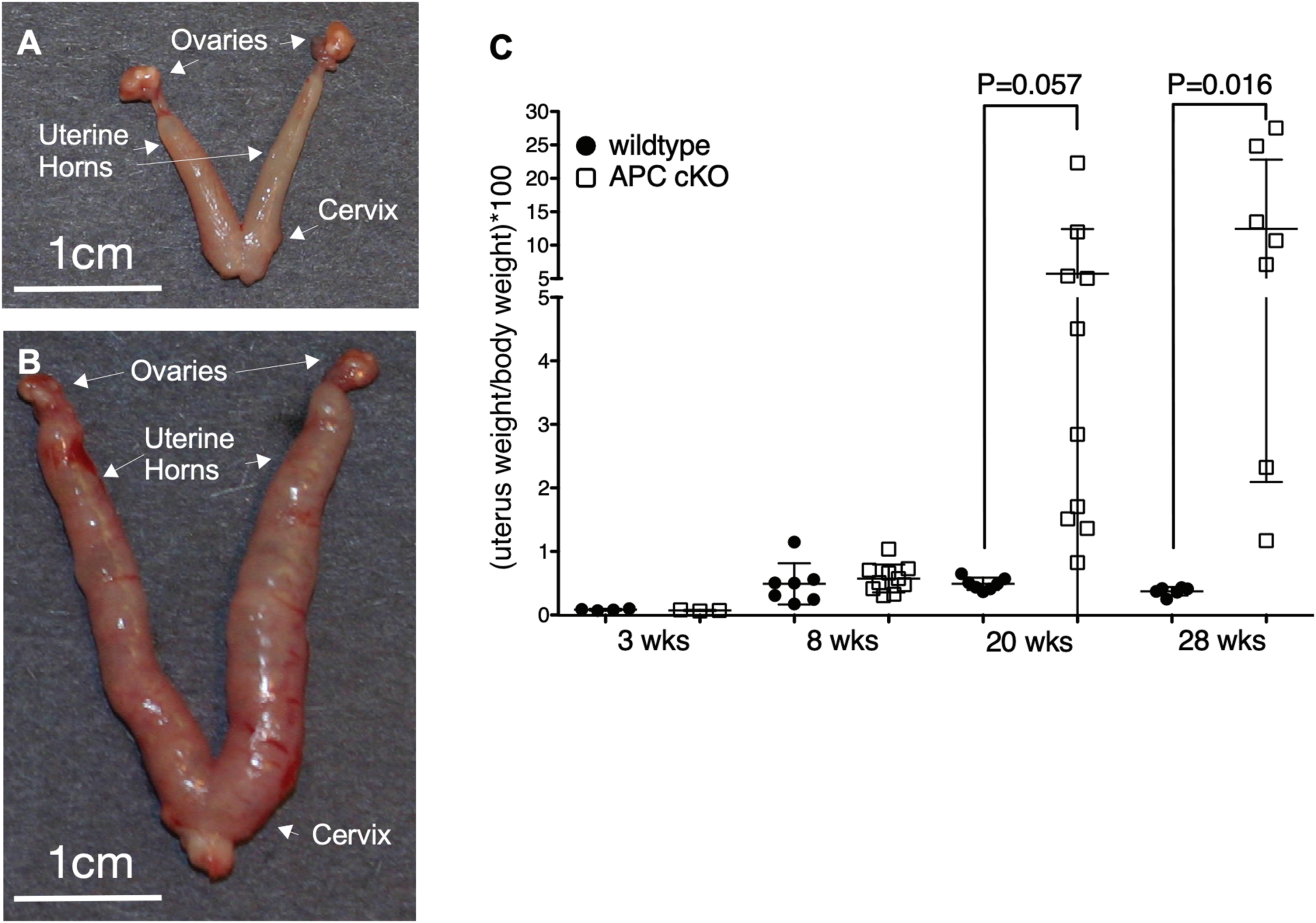
Loss of APC in mice LysM + cells results in uterine hypertrophy. Representative gross images of uteri from littermate control (**A**) and *Apc*^cKO^ (**B**) mice at 28 weeks postnatal. Uterine wet weight as a percentage of body weight for littermate control and *Apc*^cKO^ mice at the indicated ages are shown in (**C)**. Data are represented as the mean ± SD. *P*-values were generated using a two-tailed, unpaired *t*-test.

**Figure 2.**
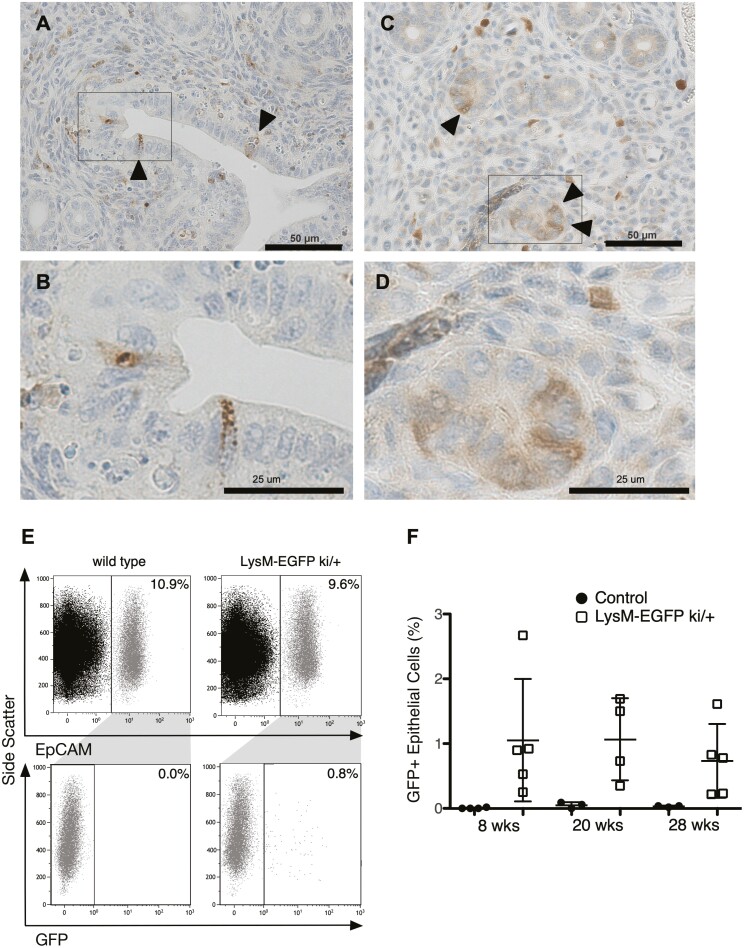
LysM is expressed in 1% of endometrial epithelial cells. Representative images of LysM IHC on 28-week-old control uterine sections showing LysM + luminal (**A**, **B**) and glandular epithelial cells (**C**, **D**). Boxed areas in A and C are shown at higher magnification in B and D. Arrowheads in A and C indicate prominently stained cells. EpCAM + endometrial epithelial cells from control and LysM-EGFP knock-in mice were analyzed by flow cytometry for GFP (**E**). Total GFP + endometrial epithelial cells from uteri of random cycling control and LysM-EGFP mice were quantitated by flow cytometry at the indicated ages (**F**). Data are represented as the mean ± SD.

We had been interested in studying how the conditional loss of APC in LysM-Cre (LysM-Cre; *Apc*^*cKO*^, hereafter called *Apc*^*cKO*^) mice, which maps to the 5q deletion region, might be involved in Myelodysplastic Syndrome. However, no obvious phenotype was observed in myeloid cells (Supplementary Figure S1, available at *Carcinogenesis* Online). Serendipitously, we found remarkable uterine hypertrophy by gross examination (Figure 1A and B) and by uterine weight by 20 weeks of age (Figure 1C) and that female mice are subfertile (Table 1). Because LysM expression has not been described in the mouse uterus, we investigated this phenomenon further. Immunohistochemistry (IHC) with an antibody that recognizes both LysM and LysP showed that in addition to macrophages that reside in the uterine stroma, a small subset of both luminal (Figure 2A and B) and glandular epithelial (Figure 2C and D) cells in the uterus express LysM. As a positive control, both the crypt cells in the intestine and the type II pneumocytes in the lung showed LysP and LysM expression, respectively (Supplementary Figure S2, available at *Carcinogenesis* Online). LysM-EGFP knock-in mice, who have EGFP knocked into the *Lyz2* locus and under the control of the *Lyz2* promoter (30), were used to verify that the lysozyme-positive EECs expressed LysM. Flow cytometry was performed on single-cell suspensions prepared from whole uterine sections of random cycling, LysM-EGFP mice to quantitate the number of LysM + EECs. Live, single, EpCAM positive cells were analyzed for GFP expression (Figure 2E and Supplementary Figure S3, available at *Carcinogenesis* Online). Approximately 1% of all EpCAM + EECs were also GFP + in the uteri of mice at 8, 20 and 28 weeks of age (Figure 2F). Taken together, these data suggest that a small percentage of EECs express LysM.

**Table 1.** LysM-Cre; *Apc*^*cKO*^ female mice are subfertile with decreased litter number per mouse and litter size

Genotype	# of mice tested	Total # of litters	Total # of pups	Mean pups/litter	Mean litters/mouse
Control	5	22	135	6	4
cKO^a^	5	5	18	4	1

Mate cages were set up with either 8-week-old control or *APC*^*cKO*^ females and allowed to mate for 6 months.

^a^cKO = LysM-cre;*Apc*^*cKO*^.

### LysM positive EECs give rise to a subset of lysozyme negative EECs

LysM-EGFP mice only express EGFP when the *Lyz2* promoter is active and might not mark daughter cells where the *Lyz2* promoter is no longer active. In order to determine whether LysM + EECs can generate daughter cells that are LysM-, LysM-Cre mice (31), which have Cre-recombinase knocked-in under the control of the *Lyz2* promoter, were crossed to a double-fluorescent reporter mouse (mT/mG) in the *Rosa26* locus that expresses membrane-targeted Tomato Red fluorescent protein prior to Cre-mediated excision and membrane-targeted EGFP after Cre-mediated excision (34). Because Cre-mediated excision is a permanent genetic event, the progeny of LysM-expressing cells will be GFP + even if the *Lyz2* promoter is no longer active.

**Figure 3.**
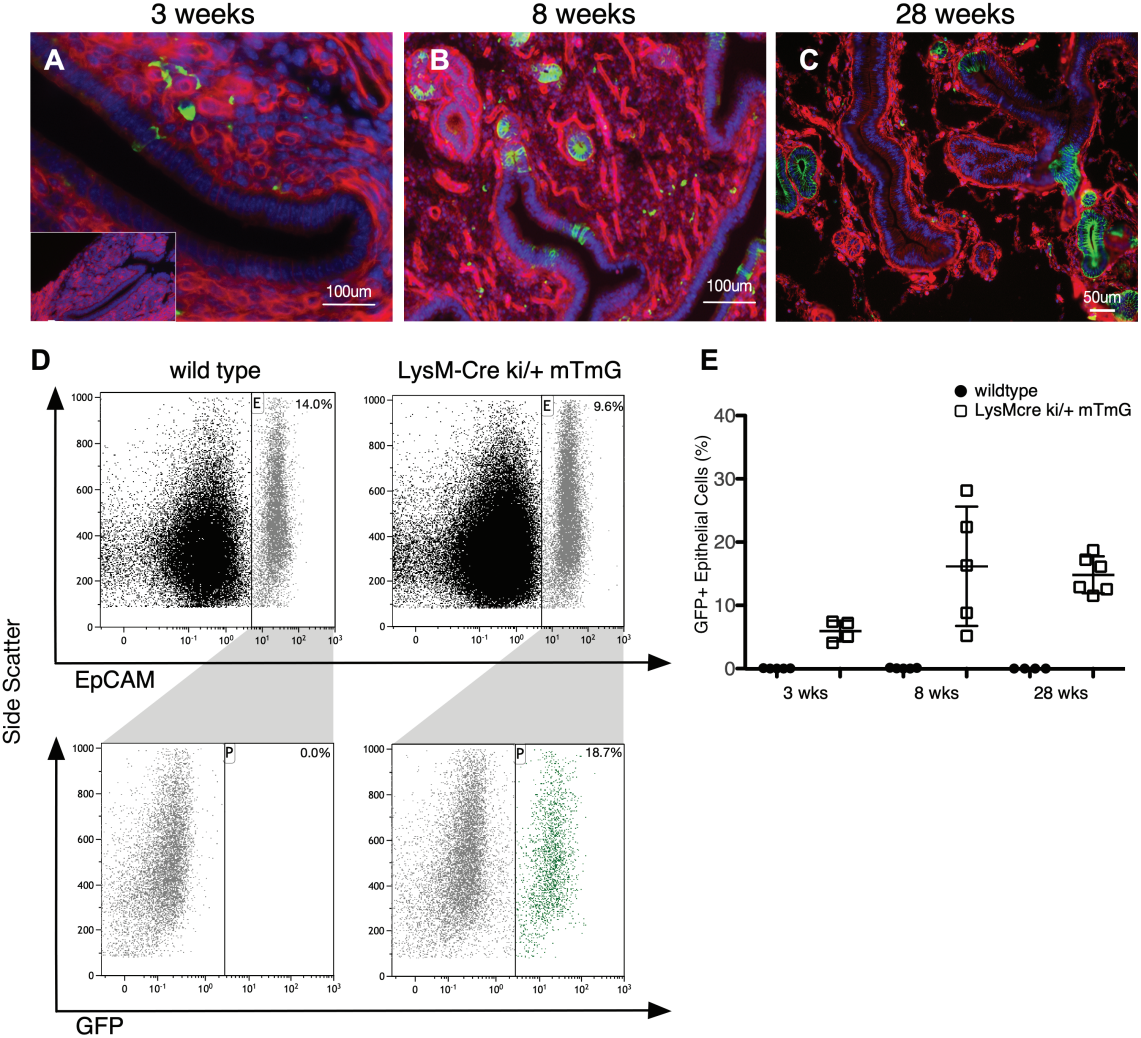
LysM + endometrial epithelial cells proliferate, and daughter cells can be LysM-. LysM-Cre mice were bred to mT/mG reporter mice to trace the lineage of uterine LysM + daughter cells. GFP + endometrial epithelial cells were observed by fluorescence microscopy in uterine sections from mice at 3 (**A**), 8 (**B**) and 28 (**C**) weeks of age. Inset in A is an image of section from a littermate control. Flow cytometry gating strategy to quantitate EpCam + endometrial epithelial cells that are GFP + in the LysM-Cre lineage (**D**). Flow cytometry analysis of EpCAM + cells in uteri from random cycling LysM-Cre;mTmG lineage mice at 3, 8 and 28 weeks of age, along with age-matched controls that lack LysM-Cre (**E**). Data are represented as mean ± SD.

We next determined when in development LysM expression is activated and examined the Müllerian ducts in mouse urogenital ridges at embryonic day (ED) 13.5 and uteri at postnatal days (PND) 7 and 21 from LysM-Cre; mT/mG mice via fluorescence microscopy. ED 13.5 is when Müllerian duct formation is complete in the mouse (35), and PND 7 and 21 are just before adenogenesis occurs in the endometrium and just before estrous cycling begins, respectively (36). GFP + cells were not observed in the Müllerian ducts at ED 13.5 or in EECs at PND 7 (Supplementary Figure S4, available at *Carcinogenesis* Online). At PND 21, there are a small number of GFP + EECs that were positioned sporadically throughout both the luminal and glandular epithelium and were typically singular and surrounded by Tomato Red + cells (Figure 3A). Therefore, *Lyz2* expression is turned on in uterine epithelial cells at some point in between PND 7 and 21. At 8 weeks of age, there were more GFP + EECs that clustered together (Figure 3B). Some glands were completely GFP+, whereas others were completely Tomato Red+. In addition, we noted instances of glands that were half GFP + and half Tomato Red+. We also looked at tissue sections from 28-week-old mice and saw similar results (Figure 3C). Flow cytometry analysis was performed on uterine single cell suspensions from random cycling LysM-Cre; mT/mG mouse uteri at 3, 8 and 28 weeks to quantitate the number of EECs derived from LysM + EECs. Live, single, EpCAM + cells were analyzed for GFP expression (Figure 3D). Approximately 5% of all EECs were GFP + at 3 weeks and approximately 15% of all the EECs were GFP + at both 8 and 28 weeks (Figure 3E). These results suggest that LysM + EECs function as progenitor cells for at least a subset of EECs that proliferate when estrous cycling begins and that these progenitors produce daughter cells that can lose LysM expression.

### Loss of APC in LysM + EECs results in endometrial hyperplasia

**Figure 4.**
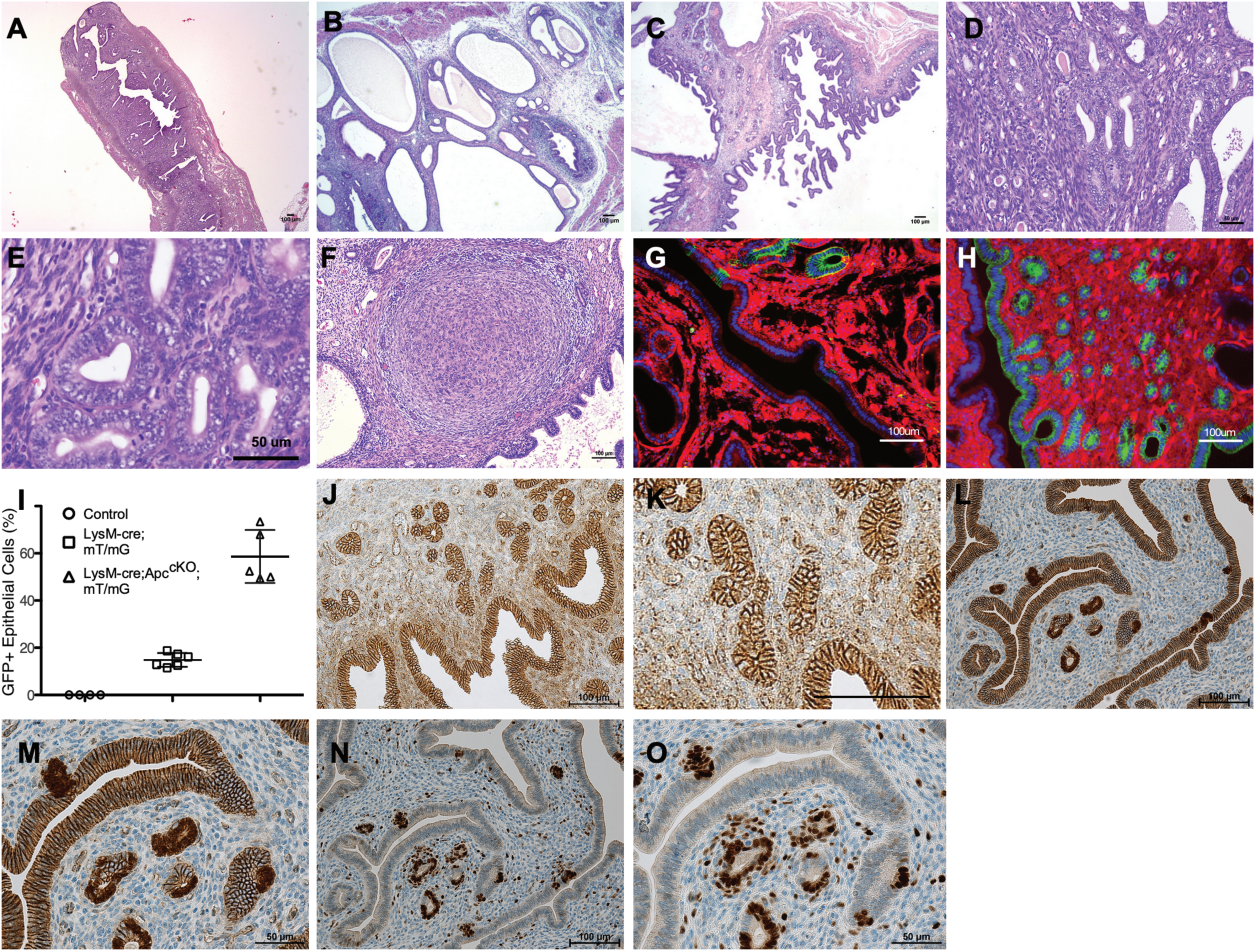
Loss of APC in LysM + endometrial epithelial cells results in endometrial hyperplasia. Representative H&E stained sections from 28-week-old control (**A**) and *Apc*^cKO^ mice demonstrating cystic endometrial hyperplasia (**B**), villoglandular or papillary hyperplasia (**C)**, complex atypical hyperplasia or EIN (**D**), nuclear atypia (**E**) and deciduoma/endometrial stroma sarcoma (**F**) histology. Images of endometrium of 28-week-old LysM-Cre; mTmG as a control (**G**) and LysM-Cre; *Apc*^cKO^; mTmG (**H**) mice. Flow cytometry analysis of GFP + cells in uteri from random cycling control (LysM-cre negative), LysM-cre; mTmG and LysM-cre; *APC*^cKO^; mTmG mice at 28 weeks postnatal (**I**). Data are represented as the mean ± SD. Representative IHC with a β-catenin antibody was performed on endometrium from control mice at 20 weeks postnatal (**J**, **K**). Representative images of IHC with β-catenin (**L**, **M**) and cyclin D1 (**N**, **O**) antibodies on uterine serial sections from an 8-week-old *Apc*^cKO^ mouse. Panels K, M and O are higher magnification images of J, L and N, respectively. Epithelial cells with nuclear β-catenin show increased expression of cyclin D1.

We showed that *Apc*^*cKO*^ mice had significantly enlarged uteri in Figure 1. In Figures 2 and 3, we showed that LysM is expressed in a subset of EECs that appear to generate daughter cells that no longer express LysM, suggesting that the uterine hypertrophy could be a result of EEC hyperplasia as a result of conditional *Apc* loss. Hematoxylin and eosin-stained sections of the uteri revealed that all of the mice examined had developed at least some form of EEC hyperplasia by 28 weeks (Table 2). At 8 weeks of age, *Apc*^cKO^ mice appear to have more glands compared to control mice and by 20 weeks of age, the glands become enlarged, cystic, and irregularly shaped compared with the control mice (Figure 4A). Cystic structures were observed throughout the uteri of *Apc*^cKO^ (Figure 4B) and the papillary lining of the lumen in some mice appeared villoglandular in structure (Figure 4C). A subset of mice at 28 + weeks of age had pathology similar to complex atypical hyperplasia (CAH)/endometrial intraepithelial neoplasia (EIN), including tightly packed glands with little to no intervening stroma, complex glandular architecture, nuclear atypia and basophilia of nuclear chromatin (Figure 4D and E, Table 2). Histological analyses of the *Apc*^cKO^ uteri also revealed numerous lesions resembling deciduomas or endometrial stromal sarcomas (Figure 4G).

**Table 2.** LysM-Cre; *APC*^*cKO*^ mice develop endometrial hyperplasia with enlarged, cystic glands

	3 weeks		8 weeks		20 weeks		28 weeks		32 + weeks	
	LC^a^	cKO^b^	LC	cKO	LC	cKO	LC	cKO	LC	cKO
Endometrial hyperplasia										
No disease	4/4	3/4	7/7	3/9	6/6	0/10	5/11	0/20	2/4	0/7
Mild	0/4	0/4	0/7	6/9	0/6	2/10	6/11	3/20	1/4	0/7
Moderate	0/4	1/4	0/7	0/9	0/6	2/10	0/11	6/20	1/4	1/7
Severe	0/4	0/4	0/7	0/0	0/6	6/10	0/11	11/20	0/4	6/7
CAH^d^/EIN^e^	N/A	N/A	N/A	N/A	N/A	N/A	0/11	6/20	0/4	3/4
Cystic										
No disease	4/4	4/4	7/7	5/9	6/6	0/10	8/11	0/20	2/4	0/7
Mild	0/4	0/4	0/7	4/9	0/6	2/10	3/11	3/20	1/4	0/7
Moderate	0/4	0/4	0/7	0/9	0/6	2/10	0/11	7/20	1/4	1/7
Severe	0/4	0/4	0/7	0/9	0/6	6/10	0/11	10/20	0/4	6/7
Deciduoma/ESS^c^										
None	4/4	4/4	7/7	9/9	6/6	10/10	11/11	14/20	4/4	7/7
Present	0/4	0/4	0/7	0/9	0/6	0/10	0/11	6/20	0/4	0/7

H&E stained sections of the female reproductive tract from control and *APC*^*cKO*^ mice at 3, 8, 20, 28 and 32 + weeks of age were assessed for the presence and severity of endometrial hyperplasia and cystic glands along with the presence of lesions resembling deciduomas or endometrial stromal sarcomas

^a^LC = Littermate Control.

^b^cKO = LysM-cre;*Apc*^*cKO*^.

^c^ESS = Endometrial Stroma Sarcoma.

^d^CAH = Complex Atypical Hyperplasia.

^e^EIN = Endometrial Intraepithelial Neoplasia.

Endometrial hyperplasia results from excess proliferation of the endometrial epithelial cells. *APC*^*cKO*^ mice were bred to mT/mG reporter mice to observe and quantitate the number of EECs in *Apc*^*cKO*^ uteri. Uterine sections from 28-week-old LysM-Cre; mT/mG control mice and LysM-cre; *Apc*^*cKO*^; mT/mG mice were analyzed by fluorescence microscopy and show that the *Apc*^*cKO*^ mice have increased GFP + EECs compared to control LysM-Cre; mT/mG uteri (Figure 4G and H). To quantitate the number of EECs in *Apc*^*cKO*^ uteri, flow cytometry analysis was performed on single cell suspensions from littermate control, LysM-Cre; mT/mG and LysM-cre; *Apc*^*cKO*^; mT/mG mice. Live, single, EpCAM + cells were analyzed for GFP expression. At 28 weeks of age, approximately 70% of all EECS were GFP + in the LysM-cre; *Apc*^*cKO*^; mT/mG mice, compared to 15% in control LysM-Cre; mT/mG mice (Figure 4I). These data suggest that loss of *Apc* in LysM + EECs leads to hyper-proliferation of EECs.

APC functions as a scaffolding component of the destruction complex that brings together multiple proteins, including GSK3β and axin, to phosphorylate β-catenin and target it for ubiquitin-mediated degradation by the proteasome. In cells lacking APC, the destruction complex does not form, and therefore β-catenin accumulates in the cytoplasm and translocates to the nucleus, where it interacts with other transcription factors to activate transcription of target genes such as *Ccnd1* and *Myc* (1). Therefore, we predicted that EECs from *Apc*^*cKO*^ would have increased levels of β-catenin and performed β-catenin IHC on uteri from 3-, 8-, 20- and 28-week-old mice. In control EECs, β-catenin is localized to the adherens junctions at the cell membrane (Figure 4J and 4K). In the *Apc*^*cKO*^ uteri, EECs lacking APC have increased levels of β-catenin in the cytoplasm and nuclear localization (Figure 4L and M). The *Ccnd1* gene is a downstream transcriptional target of WNT signaling and is a marker of nuclear β-catenin activity (37,38). IHC on serial sections shows increased cyclin D1 nuclear protein in the EECs where nuclear β-catenin was observed (Figure 4N and O). These data suggest that EECs derived from LysM + cells that are lacking APC have activated WNT signaling and increased proliferation.

### Hormone regulation of endometrial hyperplasia

**Figure 5.**
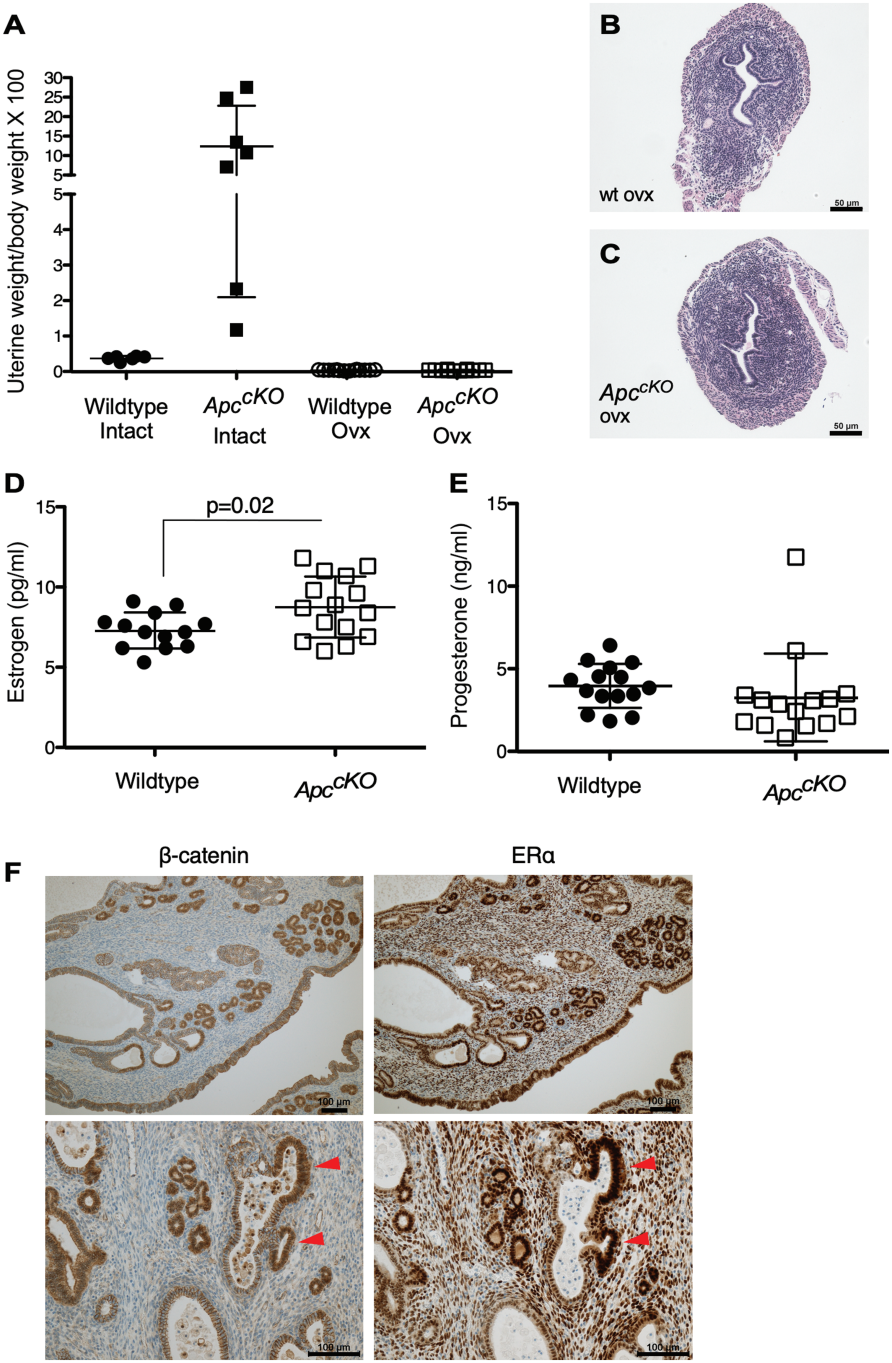
Endometrial hyperplasia in *Apc*^cKO^ mice is hormone dependent. Uterine wet weight as a percentage of total body weight for 28-week-old intact control and *Apc*^cKO^ mice compared to those from 28-week-old ovariectomized control and *Apc*^cKO^ mice as indicated (**A**). H&E stained endometrial sections from 28-week-old ovariectomized control (**B**) and *Apc*^cKO^ (**C**) mice showing similar pathology. Serum estradiol (**D**) levels are slightly elevated in the *Apc*^cKO^ mice compared to control mice. Progesterone (**E**) levels are not significantly different in control and *Apc*^cKO^ mice. Data are represented as the mean ± SD. *P*-values were generated using a two-tailed, unpaired *t*-test. (**F**) IHC with β-catenin and ER-α antibodies on serial sections from 20-week-old *Apc*^cKO^ endometrium shows that endometrial epithelial cells with increased β-catenin also have increased ER-α. Red arrowheads indicate prominent areas with excess β-catenin and ER-α expression.

We showed above that the expansion of the LysM + derived EECs occurred after 3 weeks of age (Figure 3), which is when mice begin estrous cycling (36). Ovariectomies were performed on control and *Apc*^*cKO*^ mice at 4 weeks of age to determine whether steroid hormone stimulation is necessary for the expansion of this cell population. Mice were then aged to 28 weeks before the examination. Uterine wet weights in the ovariectomized *Apc*^*cKO*^ mice were similar to that of ovariectomized control mice (Figure 5A). Additionally, hematoxylin and eosin-stained sections of the uteri reveal small, atrophic uteri for both as would be expected with a lack of hormones (Figure 5B and C). These data suggest that the LysM + EECs require hormone stimulation to proliferate. We also checked circulating estrogen and progesterone levels in the blood serum of random cycling mice. *Apc*^*cKO*^ mice have a small but statistically significant increase in estrogen levels compared to controls (Figure 5D). Progesterone levels were lower in *Apc*^*cKO*^, although not statistically significant (Figure 5E). Analysis of estrogen receptor-α (ERα) expression did show that its levels were higher by IHC in EECs where nuclear β-catenin accumulation was observed (Figure 5F), suggesting that nuclear b-catenin can induce ERα expression.

## Discussion

Our lab has identified a novel population of EECs in mice that are characterized by the expression of LysM. EECs have not been previously classified other than to categorize them as glandular or luminal. LysM has been reported in endometrial lysates but was thought to be from resident macrophages (30). Additionally, Keshav et al. reported that “no lysozyme producing epithelial cells were detected in the resting, gestating or lactating female genital tract” by in situ hybridization (39). LysM + EECs have probably gone unidentified due to the fact that at any given time, they appear to make up approximately 1% of all EECs in adult mice. We speculate that these LysM + EECs are specialized cells within the endometrium that secretes LysM into the lumen to support innate immunity in the uterus, similar to Paneth cells in the intestine (40). Innate immunity is a critical host defense mechanism in the endometrium (29), but lysozyme expression at this site has been thought to be provided by bone-marrow-derived immune cells, not the epithelial cells. Although we show only a small subset of the EECs express LysM, we speculate that residential production of LysM, rather than transient immune production, could be sufficient to help ward off bacterial infection. It would be interesting to ablate these cells specifically and determine whether the ability of the altered uteri to inhibit infection is compromised or whether they are important for embryo attachment.

Additionally, we have shown that these LysM + EECs can proliferate and give rise to approximately 15% of all EECs in our lineage tracing experiments, including LysM − EECs. This suggests LysM + EECs act as progenitor cells. Why this occurs is not clear, but it would be interesting to determine possible molecular mechanisms controlling how these progenitors contribute daughter cells to the endometrial epithelium. Most of these daughter cells do not appear to express LysM and their biological role, if any, in the endometrial epithelium is not clear.

We can only speculate on the origin of these LysM + EECs. Other labs have shown evidence that bone marrow cells can transdifferentiate into uterine epithelial cells in both human patients and mice (41,42). Bratincsak et al. also reported that CD45 + cells give rise to uterine epithelial cells and that as mice aged the number of uterine epithelial cells derived from these cells increased (42), although others have reported results that suggest otherwise (43). Our data indicate that the LysM + subset of EECs are not present before estrous cycling, suggesting that transdifferentiation begins in response to ovarian steroid hormone signaling.

The other novel finding in this study is that loss of *Apc* in EECs leads to uterine hypertrophy, secondary to endometrial hyperplasia, which we hypothesize is the result of dysregulated β-catenin nuclear activity. In human endometrial cancer, *APC* expression can be attenuated by either mutation or promoter methylation (18,19,22). Previous studies reported that expression of a constitutively activated form of β-catenin in the uterus (11–14) can lead to endometrial hyperplasia and carcinoma, but whether deletion of *Apc* in EECs alone can produce a similar phenotype has not been previously shown. Our results clearly show, for the first time, that loss of *Apc*, in at least a subset of mouse EECs, invariably leads to endometrial hyperplasia.

This mouse model is similar to human endometrial hyperplasia. We observed widespread atypical cellular and architectural features similar to what is described for EIN or atypical hyperplasia utilized in diagnosis and clinical patient management (44,45). Furthermore, atypical endometrial hyperplasia and type I endometrial cancer (EC) in humans are hormone dependent and we see this observation in our mouse model (46). The uterine hypertrophy observed in the mouse model presented is largely due to widespread cystic dilation and serous fluid accumulation. While this is not a common feature of human endometrial hyperplasia, it is common in mouse models of endometrial hyperplasia and cancer (13,47,48).

Our mouse model is unique in that we were able to knock out *Apc* in a small EEC progenitor cell population, which allowed for clonal expansion of the mutated cells. This mimics human CAH and EC, in that they have been found to have a clonal growth pattern (49). For this reason, our model could be used to study the progression of atypical endometrial hyperplasia to cancer. Follow-up studies might include crossing stabilized β-catenin or *Pten* flox mice with the LysM-cre, which represent mutations commonly observed in human type I EC. Initiating these mutations in the LysM + EEC cells might present a more relevant model for the progression of endometrial hyperplasia to EC. However, whether the human endometrium contains similar LysM + cells remains to be determined.

We believe that these findings shed light on a previously poorly understood realm of endometrial epithelial cell subtypes and their contributions to endometrial pathologies, including hyperplasia and carcinogenesis, and provide a new genetic scaffold upon which to develop future treatments and interventions.

## Supplementary Material

bgac101_suppl_Supplementary_Figures

## Data Availability

The data underlying this article are available in the article and in its online supplementary material.
