# Supplementary material for: Endometrial hyperplasia with loss of APC in a novel population of Lyz2-expressing mouse endometrial epithelial cells
Source: Carcinogenesis. 2022 Dec 22;44(1):54–64. doi: 10.1093/carcin/bgac101 (PMC10183639; doi:10.1093/carcin/bgac101)
Supplement: bgac101_suppl_Supplementary_Figures [file bgac101_suppl_supplementary_figures.docx]

**Supplemental Figures**


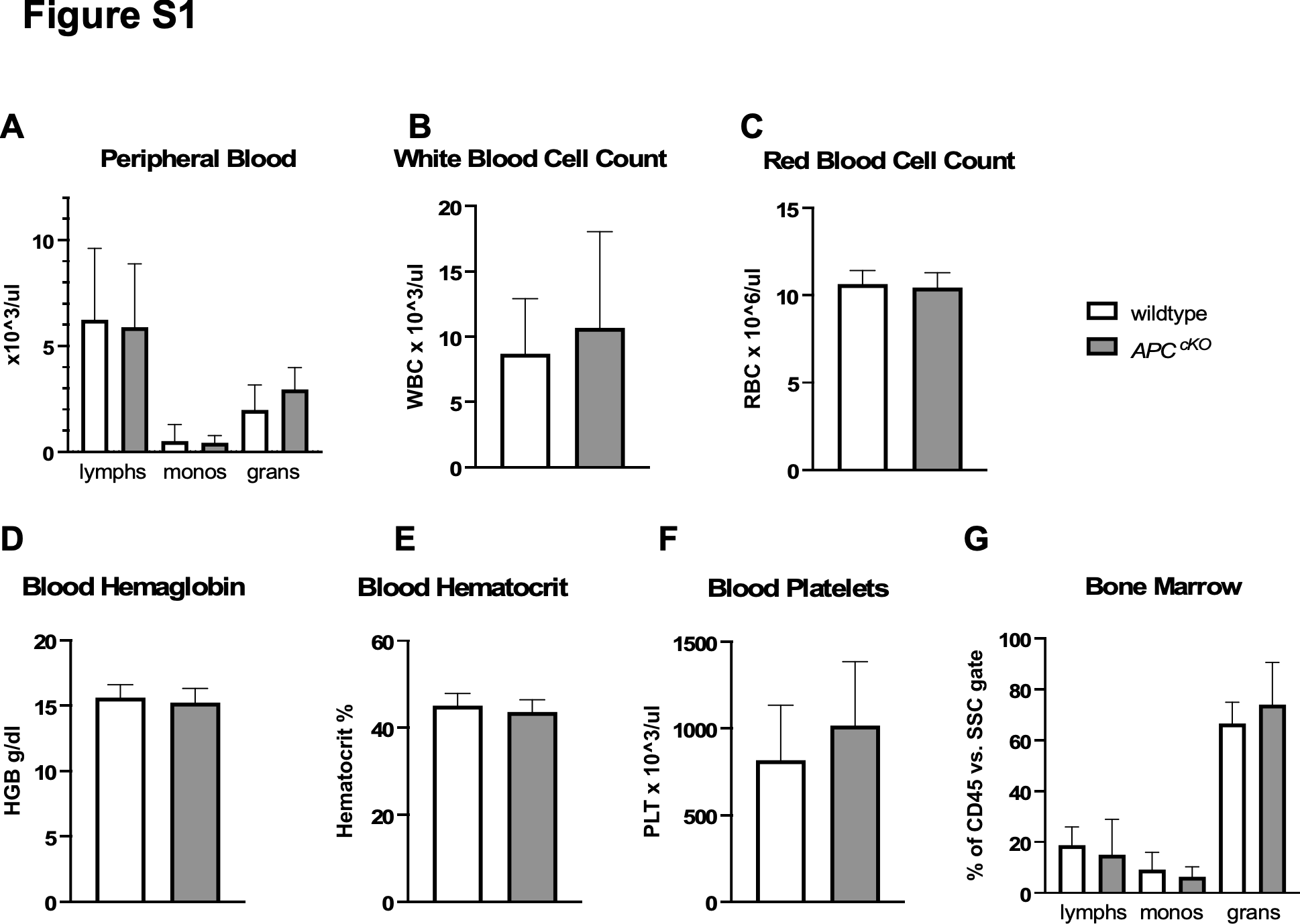


**Figure S1. Myeloid cell phenotype in LysM-Cre;*****Apc^cKO^* mice.**

Complete blood count analyses of peripheral blood in control (n=9) and *Apc^cKO^* mice (n=17) between 150-300 days postnatal (**A-F**). Bone marrow analyses of same mice by flow cytometry (**G**). No statistically significant difference between control and *Apc^cKO^* mice was observed in any of the analysis by two-tailed, unpaired student’s t test.


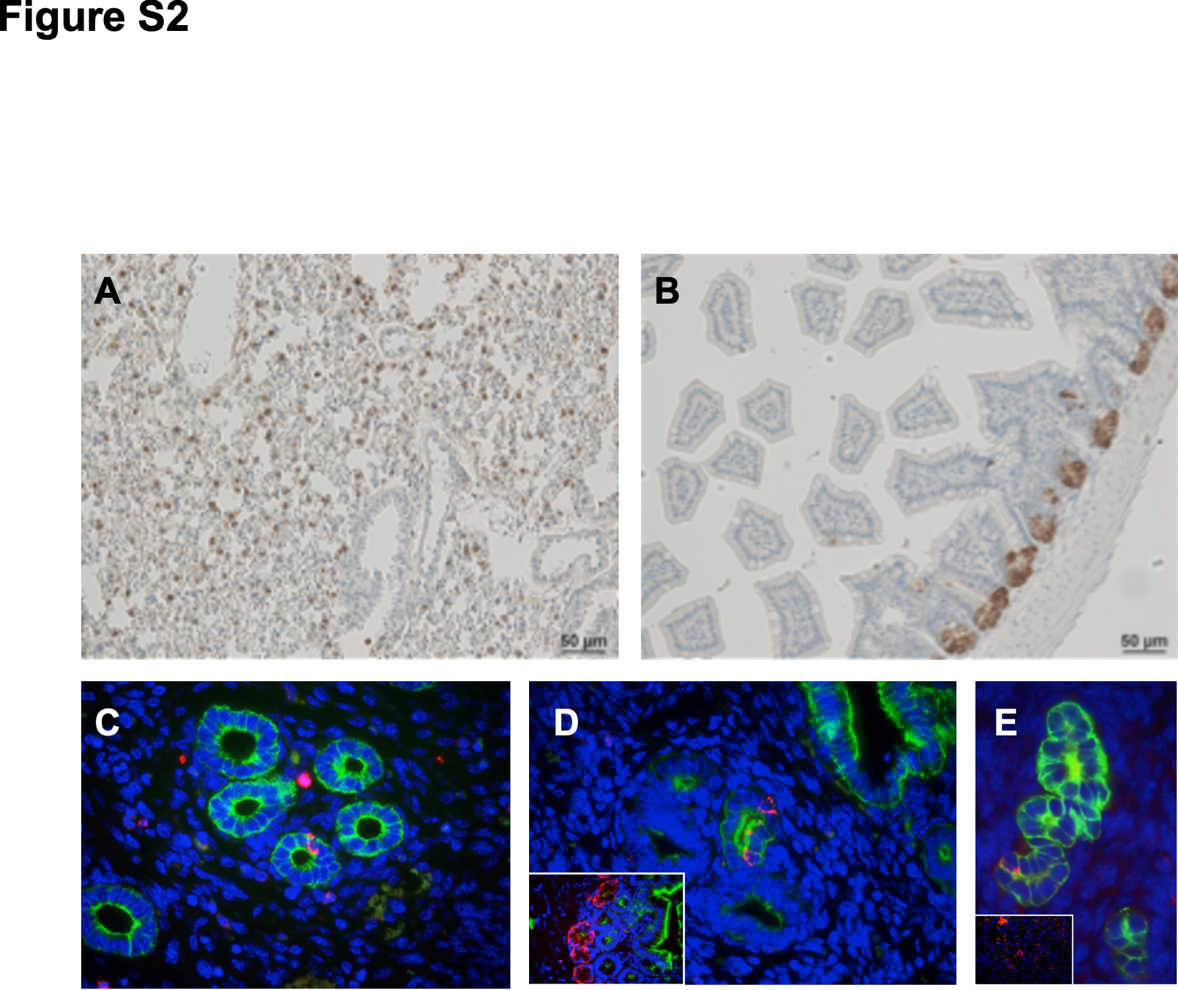


**Fig S2. Lysozyme expression.**

LysM and LysP expression is shown in type II pneumocytes (**A**) and intestinal crypt cells (**B**) by immunohistochemistry, respectively. Immunofluorescence was performed on control (**C, E**) and *Apc^cKO^* (**D**) uterine sections from postnatal day 21 (**E**) and 8 weeks postnatal (**C, D**). Lysozyme antibody in red & cytokeratin in green shows cytokeratin positive cells expressing lysozyme. Insets are positive controls (Paneth cells in intestinal crypts & type II pneumocytes in lung).


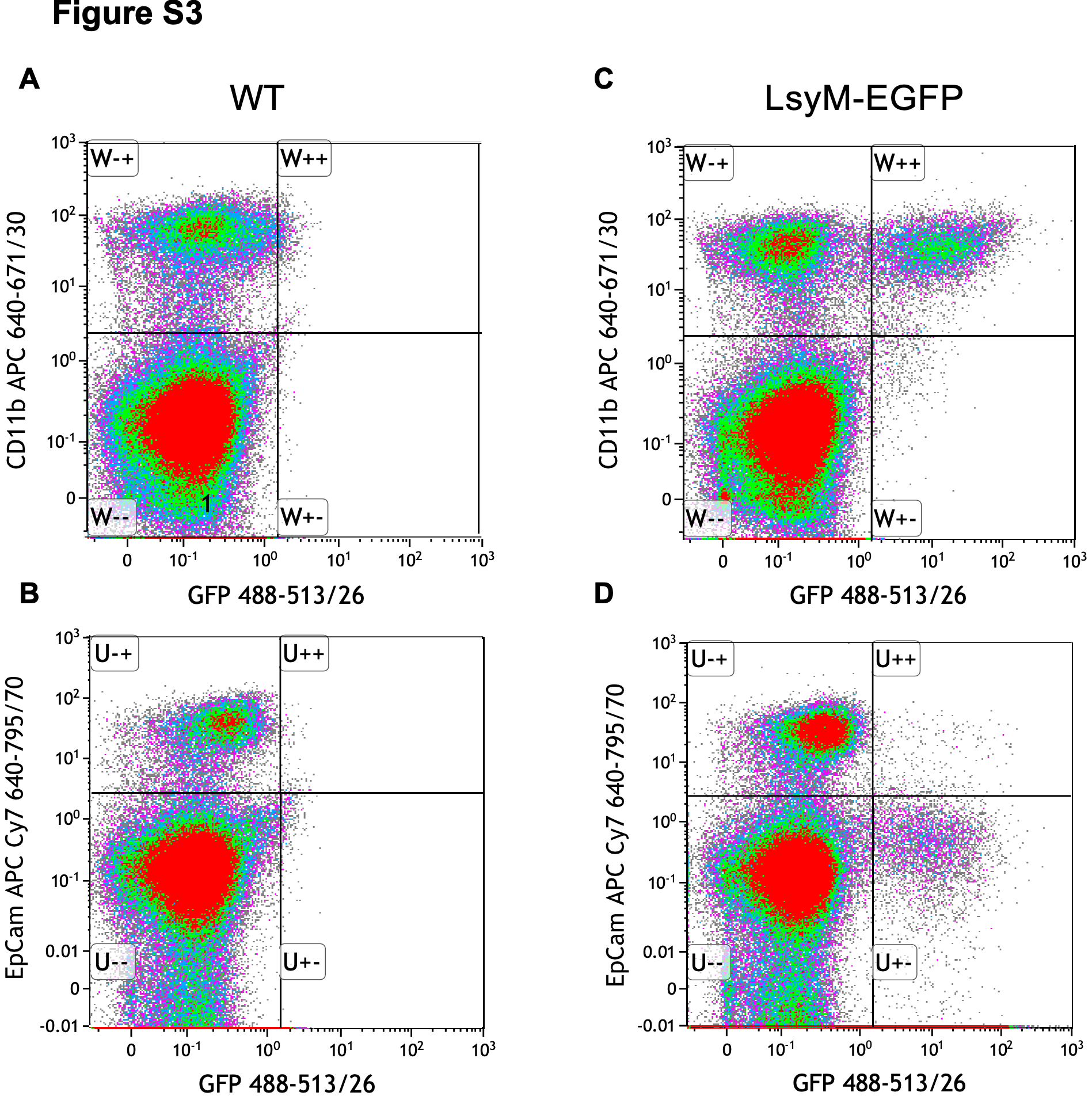


**Fig S3. Gating strategy for GFP+ endometrial epithelial cells.**

Endometrial cells were isolated from uteri of LysM-EGFP mice as analyzed by flow cytometry. Neither CD11b+ (**A**) nor EpCam+ (**B**) cells show many live GFP+ cells in uteri from control mice. Analyses of live cells from uteri of LysM-EGFP mice that were gated by CD11b to exclude myeloid lineage cells and then by GFP show few CD11b-/GFP+ cells (**C**). Analyses of live cells from uteri of LysM-EGFP mice that were gated by EpCam to identify epithelial cells and then GFP show few double positive cells (**D**).

**
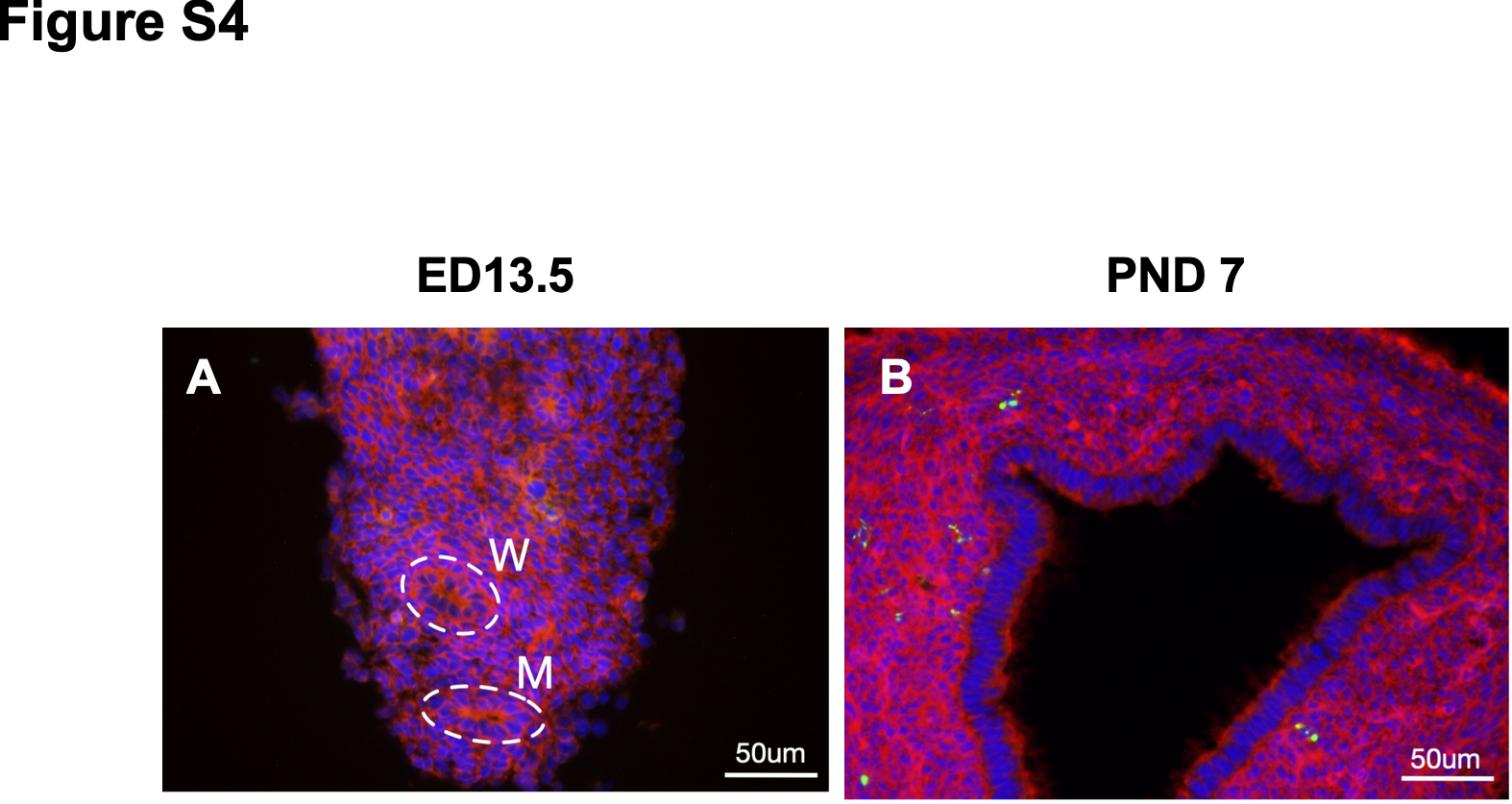
**

**Fig S4. LysM expression is absent in undifferentiated uteri of mice.**

Sections from urogenital ridges isolated from LysM-Cre;mTmG mice at embryonic day 13.5 were analyzed for GFP expression by fluorescence microscopy (**A**). At this time point, the urogenital ridges contain both the Mullerian ducts (M), which is the female reproductive tract primordia, and the Wolffian duct (W), which is the male reproductive tract primordia. Uterine section from LysM-Cre;mTmG at postnatal day 7 before adenogenesis shows GFP expression in some of the stromal cells but not the epithelial cells (**B**).
